# DEXA-Derived Body Fat Distribution and Its Correlation With Clinical, Anthropometric, Insulin Sensitivity, Metabolic, and Hormonal Parameters Among Kashmiri Polyendocrine Metabolic Ovarian Syndrome (PMOS) Women: A Cross-Sectional Study

**DOI:** 10.7759/cureus.113105

**Published:** 2026-07-21

**Authors:** Syed Douhath Yousuf, Waseem Ahmed, Ajaz Qadir, Nafeez u Rehman, Khalid u Islam, Mohd Ashraf Ganie

**Affiliations:** 1 Biochemistry, Kashmir Medical College, Srinagar, IND; 2 Endocrinology, Sher-i-Kashmir Institute of Medical Sciences, Srinagar, IND; 3 General Medicine, Kashmir Medical College, Srinagar, IND; 4 Clinical Research, Sher-i-Kashmir Institute of Medical Sciences, Srinagar, IND; 5 Endocrinology and Metabolism, Sher-i-Kashmir Institute of Medical Sciences, Srinagar, IND

**Keywords:** android fat, dexa, gynoid fat, pcos, trunk fat

## Abstract

Background: Polyendocrine metabolic ovarian syndrome (PMOS) is a common endocrine and metabolic disorder affecting women of reproductive age. It is characterized by hyperandrogenism, ovulatory dysfunction, and polycystic ovarian morphology (PCOM) and is commonly associated with obesity, particularly central adiposity, which is linked to metabolic and hormonal derangements. Dual-energy X-ray absorptiometry (DEXA) enables the precise assessment of regional body fat distribution, overcoming the limitations of conventional anthropometric indices, which inadequately characterize fat distribution.

Objectives: This study aimed to assess the body fat distribution among Kashmiri women with PMOS using DEXA (GE Lunar densitometer, GE HealthCare, Chicago, IL, USA) and further evaluate its correlation with anthropometric, metabolic, insulin sensitivity, and hormonal parameters.

Methods: This cross-sectional study included 157 women aged 18-45 years diagnosed with PMOS according to the Rotterdam criteria. Whole-body DEXA was used to assess total, android, trunk, gynoid, arm, and leg fat in PMOS women. Pearson's correlation analysis was employed to examine associations of regional fat depots with anthropometric, metabolic, insulin sensitivity, and hormonal variables.

Results: The mean age of PMOS patients was 22.83±2.82 years, with a mean body mass index (BMI) of 24.86±3.84 kg/m² and a mean waist-to-hip ratio (WHR) of 0.94±0.06. The mean total body fat percentage was 36.65±6.78%, with relatively higher proportions of android fat (42.9±9.41%) and trunk fat (40.58±7.70%) compared to peripheral depots. Android fat presented robust significant positive correlations with BMI (r=0.697), fasting insulin (r=0.65), Homeostatic Model Assessment for Insulin Resistance (HOMA-IR) (r=0.55), and WHR (r=0.497), while weak correlation was observed with cholesterol (r=0.285), triglycerides (r=0.285), two-hour glucose (r=0.235), and Ferriman-Gallwey Score (FGS) (r=0.264) (all p<0.01). Trunk fat depicted a high correlation with fasting insulin (r=0.77), HOMA-IR (r=0.62), BMI (r=0.725), and WHR (r=0.486), while a weak correlation was observed with triglycerides (r=0.297), cholesterol (r=0.293), two-hour glucose (r=0.207), and FGS (r=0.263). Gynoid, arm, and leg fat demonstrated a strong and significant correlation mainly with anthropometric parameters.

Conclusion: Women with PMOS had greater proportions of android and trunk fat, according to DEXA analysis. Also, DEXA-based fat distribution indicated significant correlation with insulin sensitivity, anthropometric, metabolic, and clinical hyperandrogenism parameters, whereas no substantial correlation patterns were observed with the hormonal variables evaluated in this study.

## Introduction

Polyendocrine metabolic ovarian syndrome (PMOS) is a prevalent endocrine disorder defined by polycystic ovarian morphology (PCOM), oligo-anovulation, and clinical and/or biochemical hyperandrogenism. It often affects 6-20% of women of reproductive age [1]. PMOS is characterized by raised inflammatory markers, dyslipidemia, elevated insulin levels, and obesity, especially abdominal obesity [2,3]. According to studies, 30-70% of women with PMOS are obese [4] and mainly present with abdominal obesity [5], which further worsens the clinical and biochemical features of PMOS [6].

The most widely used indicators for diagnosing obesity are body mass index (BMI), waist circumference (WC), and waist-to-hip ratio (WHR) [7]. Clinical and biochemical hyperandrogenism in PMOS women was found to be associated with the quantity of visceral adipose tissue and abdominal fat in a number of earlier studies [8,9]. Additionally, it was shown that women with abdominal obesity have reduced levels of sex hormone-binding globulin (SHBG), which confirms the findings that higher visceral fat storage is linked with hyperandrogenism [8]. Additionally, prior research has shown that an android-type fat distribution is linked to infertility and decreased reproductive capacity in women [10]. According to a number of studies, BMI and WC may not be enough to determine the body fat percentage. Additionally, the BMI score cannot distinguish between central and peripheral fat and does not provide information on the quantity of fat at various sites of the body [11]. Body composition analysis may provide additional information regarding regional adiposity beyond conventional anthropometric measures such as BMI and WC [12].

Although several studies have evaluated body fat distribution in women with PMOS using dual-energy X-ray absorptiometry (DEXA), data from Asian populations remain relatively limited. Therefore, in order to have a thorough understanding of the distribution of fat in PMOS patients, it is imperative to quantitatively investigate body fat deposition. An established technique for estimating total, abdominal, and extremity fat mass is a whole-body DEXA scan [13].

Aims and objectives

This study aimed to assess the distribution of body fat in Kashmiri women with PMOS using DEXA (GE Lunar densitometer, GE HealthCare, Chicago, IL, USA) and look at how this distribution relates to clinical, anthropometric, insulin sensitivity, metabolic, and hormonal parameters.

## Materials and methods

Subjects

Women with PMOS were screened and recruited from the Department of Endocrinology of a single tertiary care center, namely, Sher-i-Kashmir Institute of Medical Sciences (SKIMS) in Srinagar, Jammu and Kashmir, India, from February 2021 to February 2024. The study included 157 PMOS women aged 18-45 years. Eligible women who attended the Department of Endocrinology during the study period and met the predefined inclusion and exclusion criteria were recruited consecutively until the required sample size of 157 participants was achieved. The Rotterdam criteria [14] was used for diagnosis, requiring two out of three specific symptoms, (1) oligomenorrhea or anovulation (fewer than eight cycles in the previous year or no periods for six months), (2) clinical and/or biochemical evidence of hyperandrogenism, and (3) PCOM (12 or more follicles of 2-9 mm diameter or ovarian volume >10 cm³), after excluding other conditions such as non-classical congenital adrenal hyperplasia, androgen-secreting tumors, Cushing's syndrome, thyroid dysfunction, and hyperprolactinemia through appropriate investigations. The study also excluded women with diabetes mellitus and renal, hepatic, or cardiac problems, as well as those who had recently taken hormones or drugs that may have an impact on hormonal or metabolic processes.

**Figure 1:**
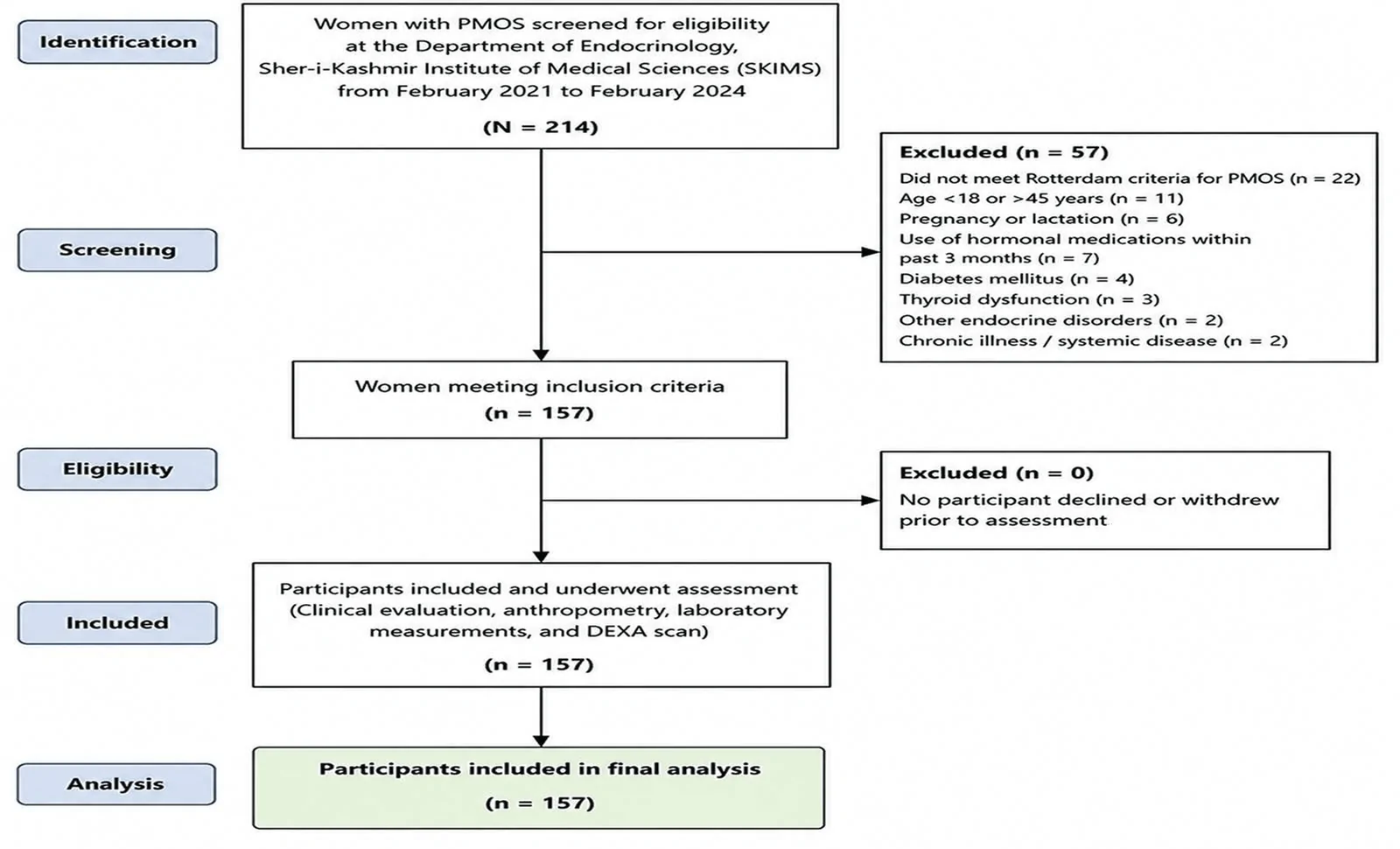
Flow diagram of participant recruitment, screening, eligibility assessment, inclusion, and final analysis. PMOS: polyendocrine metabolic ovarian syndrome; DEXA: dual-energy X-ray absorptiometry

A total of 214 women with suspected PMOS were screened for eligibility. Fifty-seven participants were excluded, primarily due to not fulfilling the Rotterdam diagnostic criteria (n=22), age outside the eligible range (n=11), pregnancy or lactation (n= 6), recent hormonal medication use (n=7), diabetes mellitus (n=4), thyroid dysfunction (n=3), other endocrine disorders (n=2), and chronic systemic illnesses (n=2). Consequently, 157 women met the eligibility criteria, and none declined participation or withdrew before assessment. All 157 eligible participants completed the clinical evaluation, anthropometric measurements, laboratory investigations, and DEXA scan and were subsequently included in the final statistical analysis, with no loss or missing outcome data (Figure 1).

The present study was conducted as a part of the multicenter PMOS Taskforce Study entitled "Prevalence, regional phenotypic variation, co-morbidities and risk factors among Indian women with PMOS and their response to different therapeutic modalities", under the Department of Endocrinology of SKIMS. The study received ethical approval from the Institutional Ethics Committee of SKIMS (approval number: 107/2016).

Anthropometric and clinical assessment

Height, weight, WHR, and blood pressure of each woman were measured. WC was measured at the narrowest portion of the torso approximately midway between the lowest rib and the iliac crest; hip circumference (HC) was measured over the widest portion of the gluteal and greater trochanteric region in the standing position, and accordingly, WHR was calculated. Anthropometric measurements were obtained using standardized procedures under uniform conditions by trained personnel.

The details of the menstrual history were recorded, along with the existence of hyperandrogenic symptoms such as hirsutism, acne, androgenic alopecia, and acanthosis nigricans. Hirsutism was measured using the Ferriman-Gallwey Score (FGS), which assigned numbers to nine different body parts [15]. Out of 36, a count of greater than 8 was considered significant.

Biochemical analysis

Following a 10- to 12-hour overnight fast, the patient had an oral glucose tolerance test (OGTT), receiving 75 g of anhydrous glucose diluted in 300 mL of water [16]. Samples of blood were drawn at zero, 60, and 120 minutes. Additional biochemical analytical examinations included tests for liver function, kidney function, insulin levels, as well as lipid profiles.

Calculations

The fasting glucose-to-insulin ratio (FGIR), the Quantitative Insulin Sensitivity Check Index (QUICKI), and the Homeostatic Model Assessment for Insulin Resistance (HOMA-IR) index were utilized to measure insulin resistance. To calculate the FGIR values, fasting glucose (mg/dL)/fasting insulin (µIU/mL) was utilized. QUICKI was calculated as 1/(log fasting insulin (µIU/mL) + log fasting glucose (mg/dL). The HOMA-IR index was calculated as follows: fasting insulin (µIU/mL) × fasting glucose (mg/dL)/405 [17,18]. Body weight (kg) divided by body height squared (m^2^) yielded the BMI.

Hormonal analysis

Non‑classical congenital adrenal hyperplasia, hypothyroidism, prolactinoma, hypergonadotropism, Cushing's syndrome, hyperandrogenism, and androgen‑secreting tumors were ruled out by performing the following investigations which included 17-hydroxyprogesterone (17‑OHP), thyroxine (T4) and thyroid-stimulating hormone (TSH), prolactin (PRL), luteinizing hormone (LH) and follicle-stimulating hormone (FSH), cortisol 8 am with 1 mg overnight dexamethasone suppression test (ONDST), total serum testosterone, and dehydroepiandrosterone sulfate (DHEAS), respectively. The third to seventh days of a progesterone-induced or spontaneous menstrual cycle was preferably used to collect the LH, FSH, 17-OHP, and testosterone samples.

Ultrasonography

Days 2-7 of the menstrual cycle were best suited for the ultrasound scan (Esaote MyLab 9, Genova, Italy). Because of this, no follicle that is growing can conceal a smaller one or change the volume of the ovary. Women who were oligo- or amenorrheic may have it done at random or 2-7 days following progesterone-induced bleeding.

Laboratory analysis

An autoanalyzer (DiaSys 200, DiaSys Diagnostics India Pvt Ltd, Navi Mumbai, India) was utilized to measure the glucose and other biochemical markers. Using commercial kits and adhering to supplier protocol, hormonal assays were carried out in duplicate by chemiluminescent immunosorbent assay on Cobas e411 (Roche Diagnostics, Mannheim, Germany). The fasting insulin levels were measured by electrochemiluminescence (Cobas e411, Roche Diagnostics).

DEXA

DEXA was performed using a GE Lunar densitometer. Participants were scanned according to standard manufacturer recommendations and were instructed to remove all metallic objects prior to the examination. The DEXA scanner was calibrated in accordance with the manufacturer's protocol prior to participant assessments, and routine quality control procedures were performed at regular intervals using standard calibration phantoms. All measurements were obtained using the same scanner under standardized conditions and analyzed by trained personnel to ensure the consistency and reliability of body composition estimates.

The body fat composition was assessed for the trunk, android, limbs, and gynoid regions along with total body fat percentage. Regional adiposity measures, including total body fat, android fat, trunk fat, gynoid fat, arm fat, and leg fat, were expressed as percentage fat (%) rather than absolute fat mass (kg), and these percentage-based measures were used for subsequent analysis. The percentage of total body fat was calculated by the ratio of total fat mass/total body mass.

Sample Size Calculation

The sample size was calculated using the nMaster 2.0 software (Department of Biostatistics, Christian Medical College, Vellore, India), with an 80% power and a 95% confidence level.

Statistical analysis

The statistical interpretation was conducted using SPSS Statistics for Windows, V. 16.0 (SPSS Inc., Chicago, IL, USA). Data obtained was expressed as mean±SD. Pearson's correlation test was used to measure the strength of the linear relationship between different variables.

## Results

A total of 157 women with PMOS were included in the final analysis. The mean age of the study population was 22.83±2.82 years. The mean body weight was 62.76±5.3 kg, with a mean BMI of 24.86±3.84 kg/m². The mean WHR was 0.94±0.06. The mean FGS was 10.07±3.89, indicating significant clinical hyperandrogenism (Table 1).

**Table 1:** Clinical and anthropometric parameters of PMOS cases. FGS: Ferriman-Gallwey Score; BMI: body mass index; WC: waist circumference; HC: hip circumference; WHR: waist-to-hip ratio; SBP: systolic blood pressure; DBP: diastolic blood pressure; PMOS: polyendocrine metabolic ovarian syndrome

Variables	Cases (n=157)	
	Mean	SD
Age (year)	22.83	2.82
FGS	10.07	3.89
Menstrual cycles/per year	7	4.9
Height (cm)	158.51	6.7
Weight (kg)	62.76	5.3
BMI (kg/m^2 ^)	24.86	3.84
WC (cm)	91	6.8
HC (cm)	96	4.2
WHR	0.94	0.06
SBP (mmHg)	114.28	13.3
DBP (mmHg)	76.7	7.86

The mean fasting glucose and two-hour post-load glucose were 88.12±5.3 mg/dL and 100.7±8.73 mg/dL, respectively. The mean fasting insulin level was 14.60±5.2 µIU/mL. Indices of insulin sensitivity demonstrated a mean HOMA-IR of 3.48±4.08, QUICKI of 0.32±0.02, and FGIR of 7.3±5.6. Lipid profile analysis revealed a mean total cholesterol of 159.9±31.3 mg/dL and triglycerides of 137.4±32.9 mg/dL. The mean LH, mean FSH, and mean testosterone levels of the PMOS group were 9.39±4.3 IU/L, 6.34±2.7 IU/L, and 65.3±6.6 ng/dL, respectively (Table 2).

**Table 2:** Serum biochemical and hormone levels of PMOS cases. HOMA-IR: Homeostatic Model Assessment for Insulin Resistance; FGIR: fasting glucose-to-insulin ratio; QUICK: Quantitative Insulin Sensitivity Check Index; HDL: high-density lipoprotein; LDL: low-density lipoprotein; LH: luteinizing hormone; FSH: follicle-stimulating hormone; PMOS: polyendocrine metabolic ovarian syndrome

Variables	Cases (n=157)	
	Mean	SD
Blood glucose, fasting (mg/dL)	88.12	5.3
Blood glucose, 2 hour (mg/dL)	100.7	8.73
Insulin fasting (µIU/mL)	14.60	5.2
HOMA-IR	3.48	4.08
FGIR	7.3	5.6
QUICKI	0.32	0.02
Cholesterol (mg/dL)	159.9	31.3
Triglycerides (mg/dL)	137.4	32.9
HDL cholesterol (mg/dL)	47.4	2.16
LDL cholesterol (mg/dL)	94	8.68
LH (IU/L)	9.39	4.3
FSH (IU/L)	6.34	2.7
Testosterone (ng/dL)	65.3	6.6

The mean total body fat percentage of PMOS patients was 36.65±6.78%, gynoid fat percentage was 38.65±4.48%, arm fat percentage was 38.13±5.54%, and leg fat percentage was 37.24±4.01%, with relatively higher proportions of mean android fat (42.9±9.41%) and trunk fat (40.58±7.70%) (Table 3).

**Table 3:** Fat mass distribution of body compartments of PMOS women. PMOS: polyendocrine metabolic ovarian syndrome

Variables (%)	Cases (n=157)	
	Mean	SD
Total body fat	36.65	6.78
Android fat	42.9	9.41
Gynoid fat	38.65	4.48
Arm fat	38.13	5.54
Leg fat	37.24	4.01
Trunk fat	40.58	7.70

**Figure 2:**
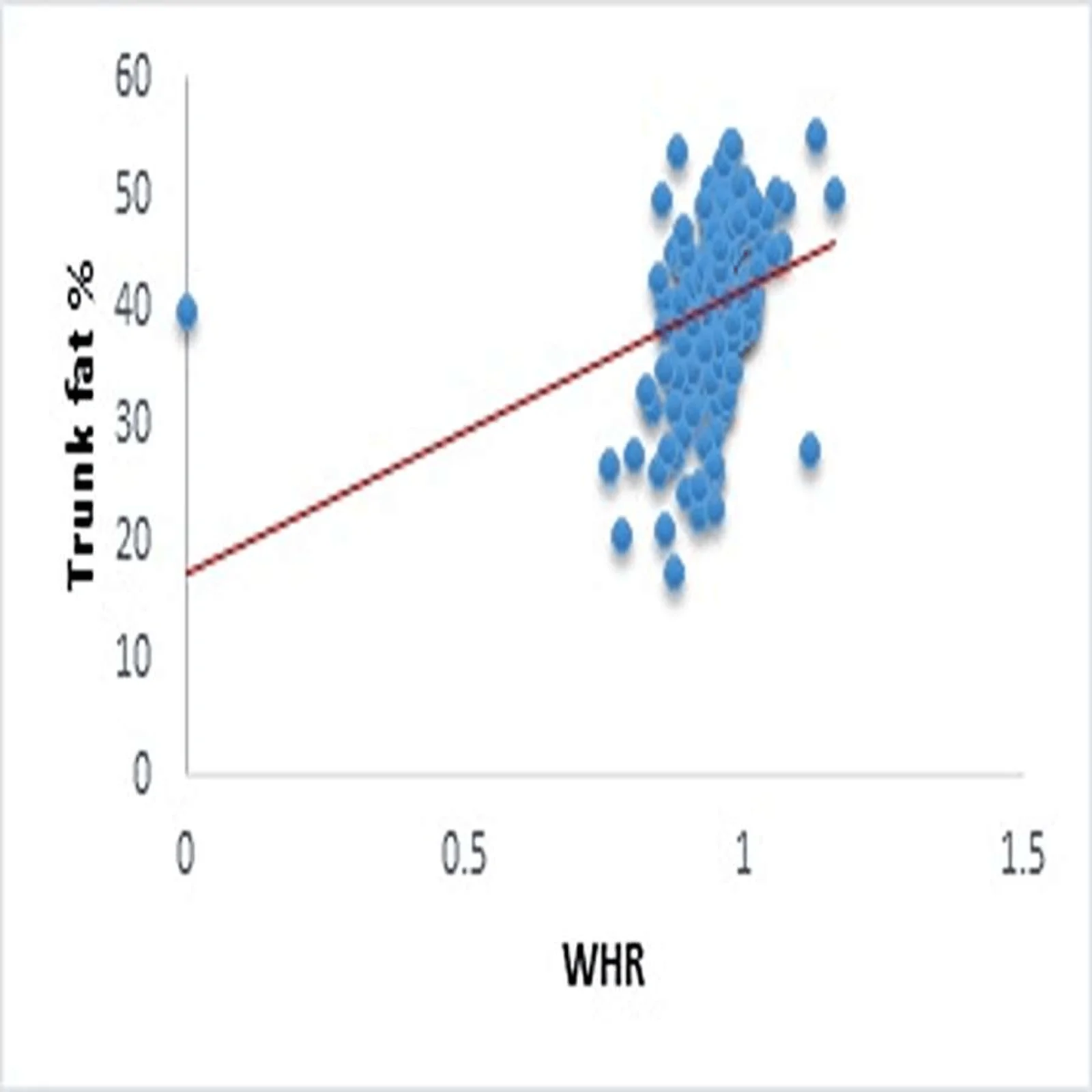
Graph showing the correlation between WHR and trunk fat (%). WHR: waist-to-hip ratio

**Figure 3:**
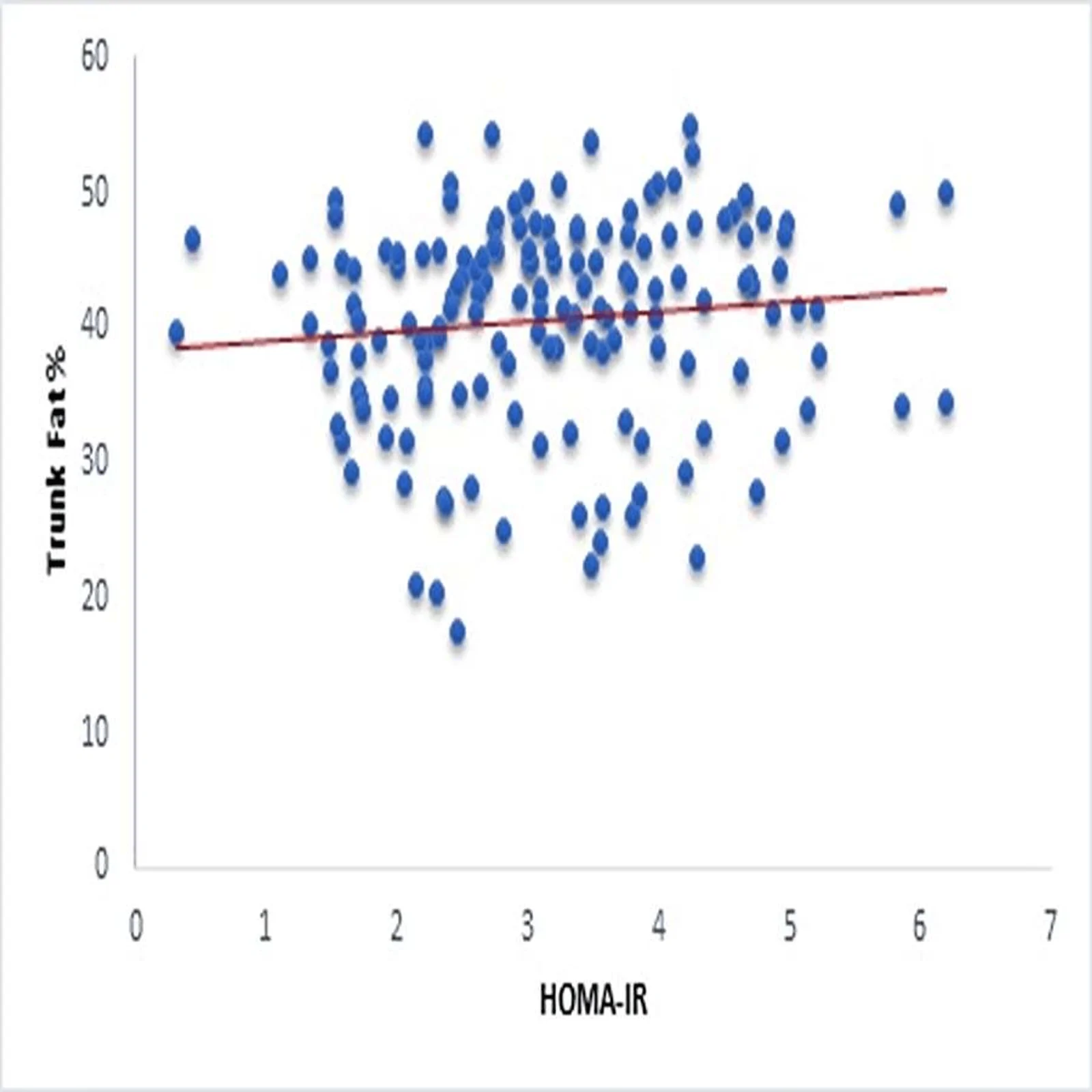
Graph showing the correlation between HOMA-IR and trunk fat (%). HOMA-IR: Homeostatic Model Assessment for Insulin Resistance

**Figure 4:**
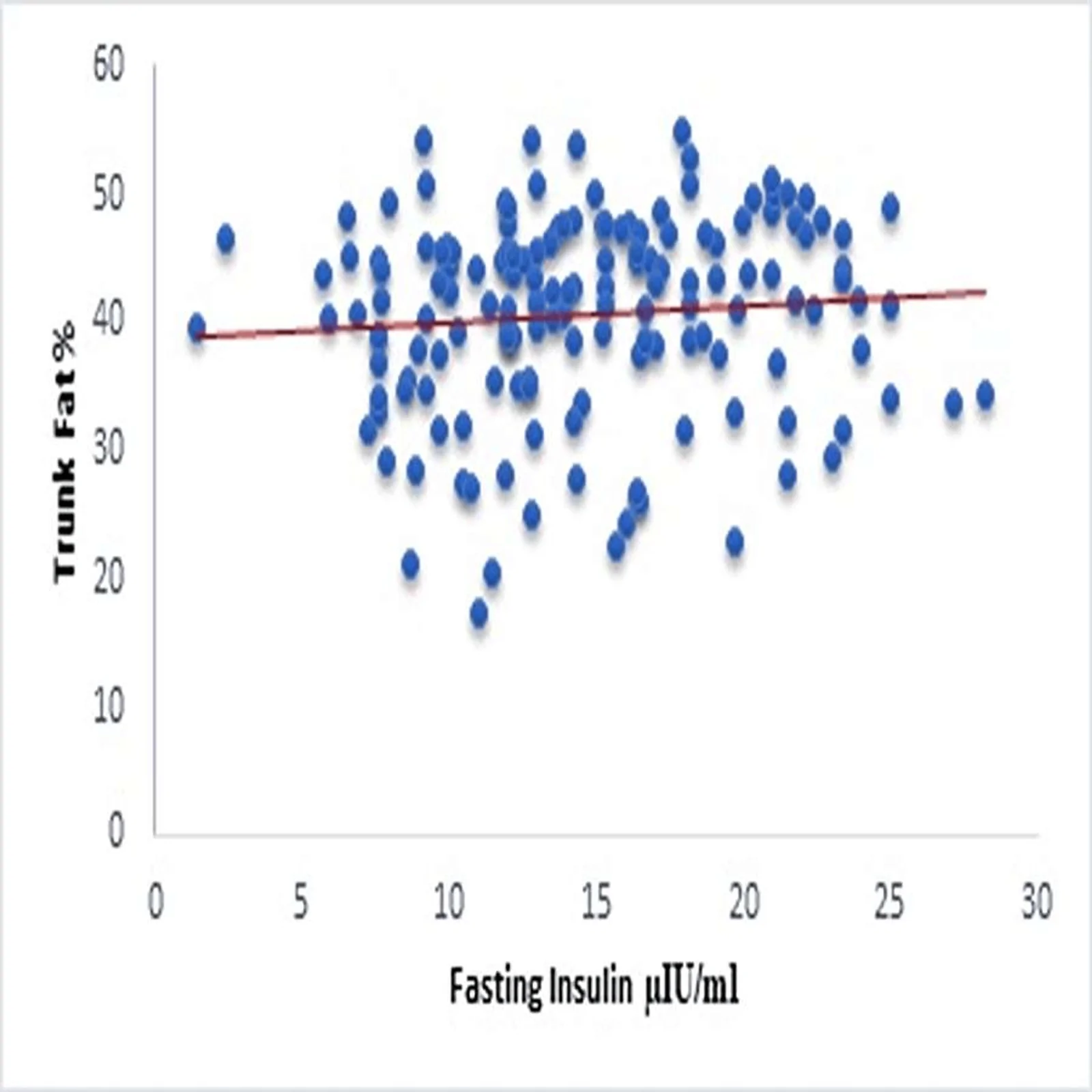
Graph showing the correlation between fasting insulin (µIU/mL) and trunk fat (%).

**Figure 5:**
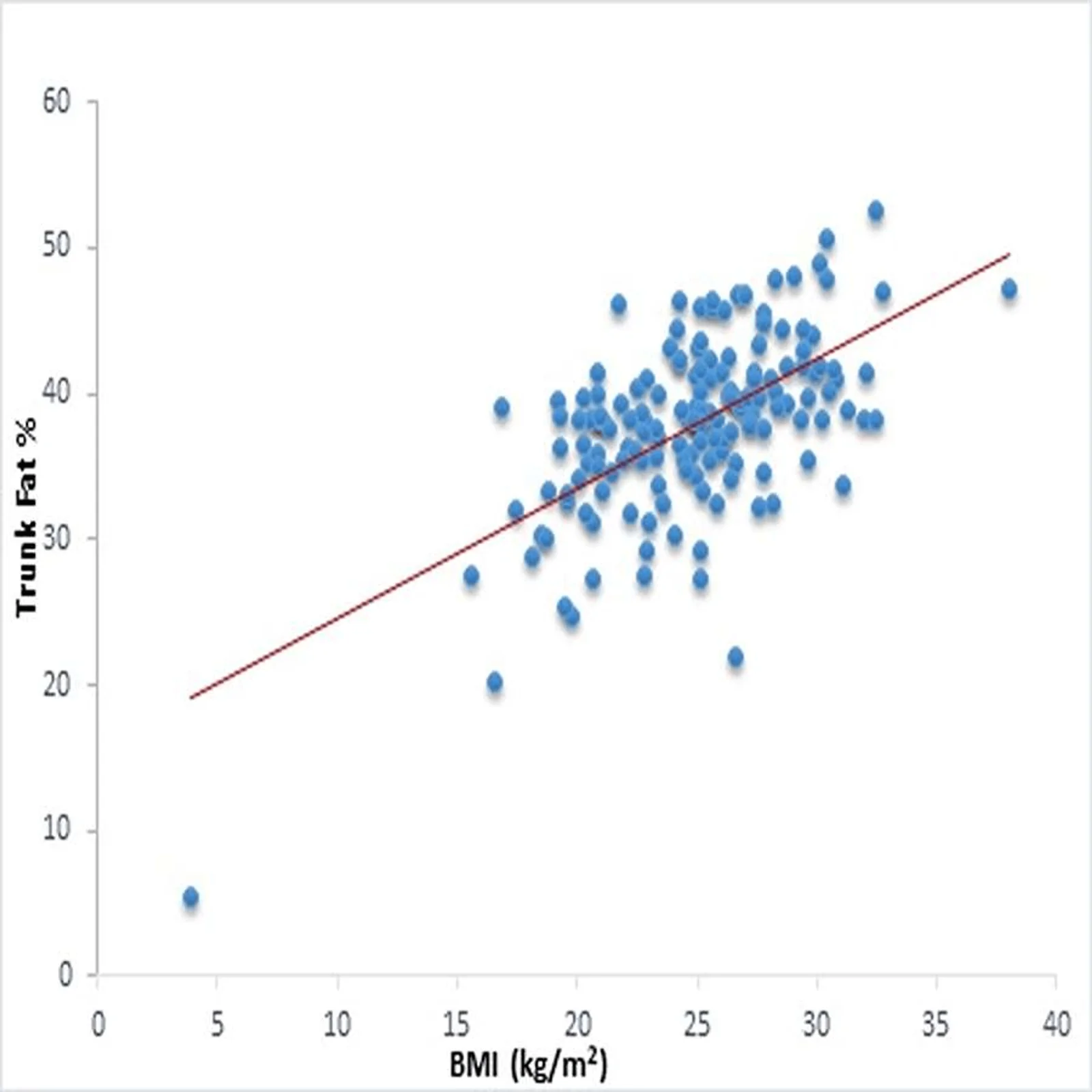
Graph showing the correlation between BMI (kg/m2) and trunk fat (%). BMI: body mass index

Trunk fat correlated significantly with fasting insulin (r=0.77; p=0.02), BMI (r=0.725; p<0.001), HOMA-IR (r=0.62; p=0.01), and WHR (r=0.486; p<0.001); also, weak correlation was observed with triglycerides (r=0.297; p=0.01), cholesterol (r=0.293; p=0.01), two-hour glucose (r=0.207; p=0.01), and FGS (r=0.263; p=0.01). Trunk fat did not demonstrate a statistically significant association with serum testosterone levels (r=0.075; p=0.34) (Table 4; Figure 2, Figure 3, Figure 4, and Figure 5).

**Table 4:** Correlation of regional fat depots with metabolic and clinical parameters in PMOS BMI: body mass index; WHR: waist-to-hip ratio; FGS: Ferriman-Gallwey Score; HOMA-IR: Homeostatic Model Assessment for Insulin Resistance; LH: luteinizing hormone; FSH: follicle-stimulating hormone; PMOS: polyendocrine metabolic ovarian syndrome

Variable	Android fat (r, p)	Trunk fat (r, p)	Total body fat (r, p)	Arm fat (r, p)	Gynoid fat (r, p)	Leg fat (r, p)
BMI (kg/m^2^)	0.697, <0.001	0.725, <0.001	0.519, <0.001	0.536, <0.001	0.521, <0.001	0.404, <0.001
WHR	0.497, <0.001	0.486, <0.001	0.262, <0.001	0.358, <0.001	0.078, 0.08	0.076, 0.09
2-hour glucose (mg/dL)	0.235, 0.003	0.207, 0.01	0.093, 0.23	0.083, 0.33	0.073, 0.29	0.088, 0.33
Triglycerides (mg/dL)	0.285, 0.001	0.297, 0.01	0.066, 0.43	0.078, 0.55	0.079, 0.79	0.080, 0.45
Cholesterol (mg/dL)	0.285, 0.001	0.293, 0.01	0.078, 0.56	0.089, 0.98	0.078, 0.87	0.087, 0.77
FGS	0.264, 0.001	0.263, 0.01	0.217, 0.006	0.066, 0.89	0.078, 0.55	0.067, 0.56
Testosterone (ng/dL)	0.061, 0.45	0.075, 0.34	0.09, 0.22	0.058, 0.47	0.019, 0.811	0.04, 0.59
Fasting insulin (µIU/mL)	0.65, 0.01	0.77, 0.02	0.49, 0.01	0.043, 0.56	0.067, 0.98	0.076, 0.88
HOMA-IR	0.55, 0.02	0.62, 0.01	0.076, 0.33	0.078, 0.77	0.087, 0.67	0.099, 0.66
LH (IU/L)	0.043, 0.55	0.056, 0.67	0.045, 0.65	0.067, 0.56	0.056, 0.45	0.067, 0.56
FSH (IU/L)	0.054, 0.65	0.067, 0.87	0.056, 0.76	0.073, 0.43	0.076, 0.55	0.075, 0.67

**Figure 6:**
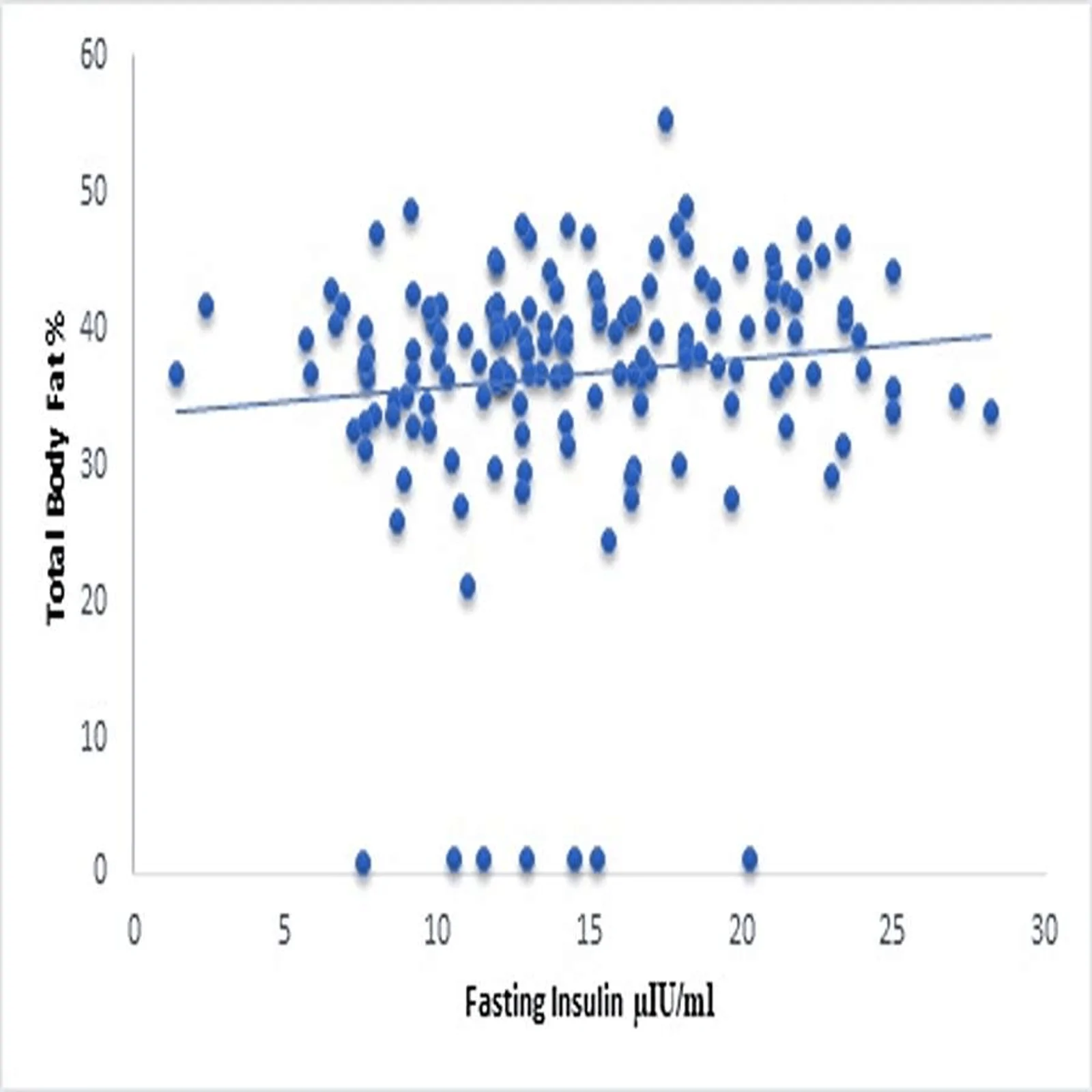
Graph showing the correlation between fasting insulin (µIU/mL) and total body fat (%).

**Figure 7:**
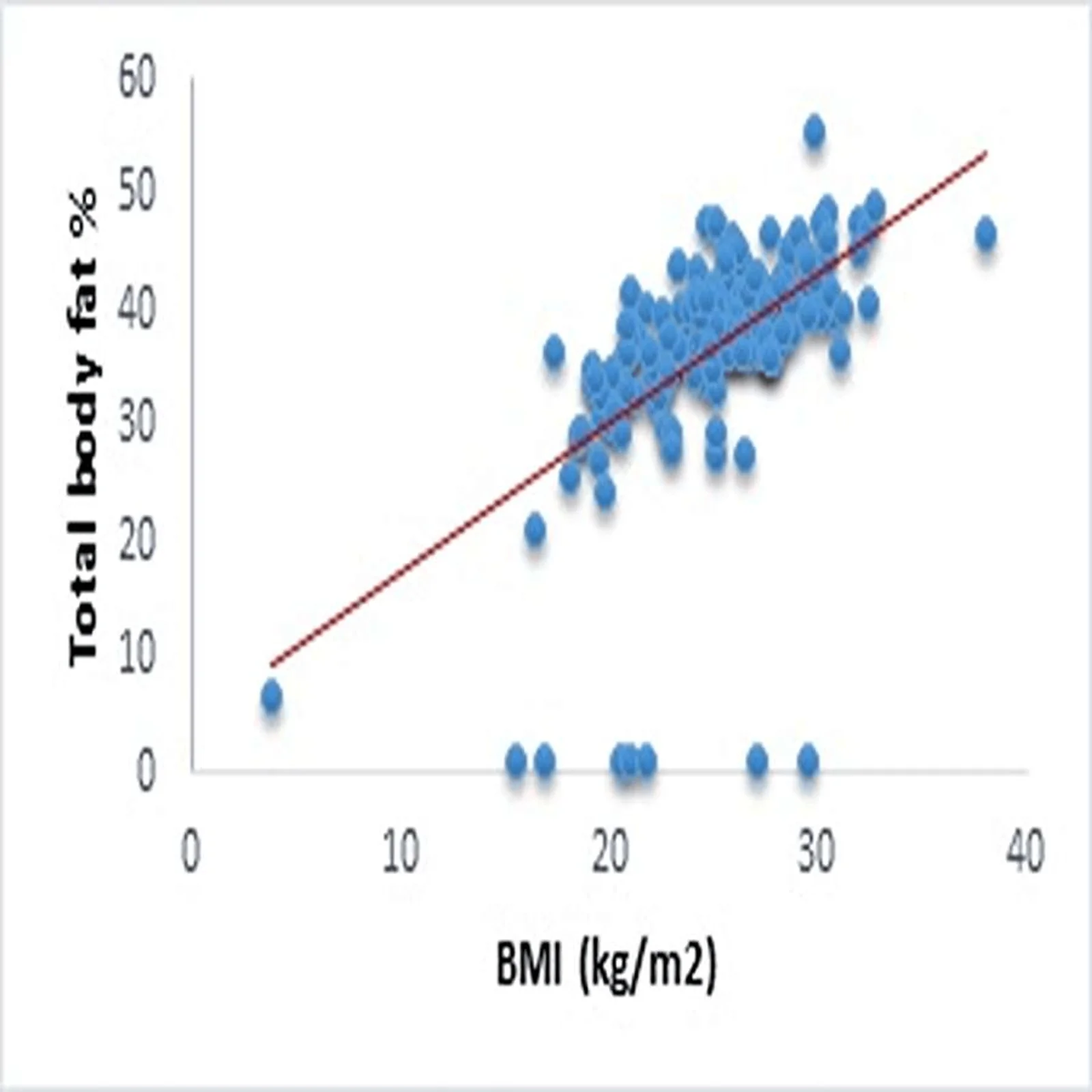
Graph showing the correlation between BMI (kg/m2) and total body fat (%). BMI: body mass index

Total body fat correlated significantly with BMI (r=0.519; p<0.001) and fasting insulin (r=0.49; p=0.01), but a weak significant association was observed with WHR (r=0.262; p< 0.001) and FGS (r=0.217; p=0.006). In contrast, total body fat did not demonstrate a statistically significant association with serum testosterone levels (r=0.09; p=0.22) (Table 4; Figure 6 and Figure 7).

**Figure 8:**
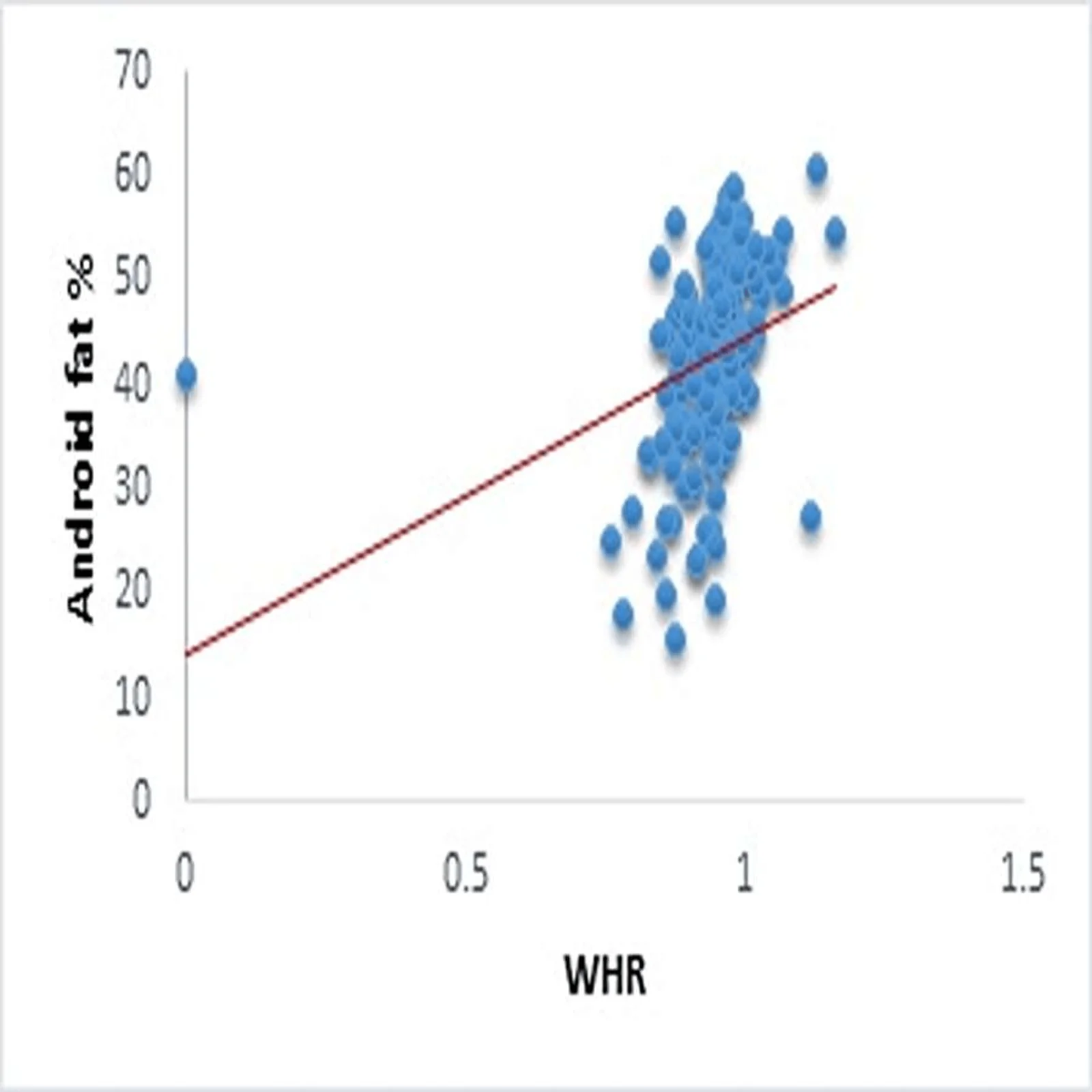
Graph showing the correlation between WHR and android fat (%). WHR: waist-to-hip ratio

**Figure 9:**
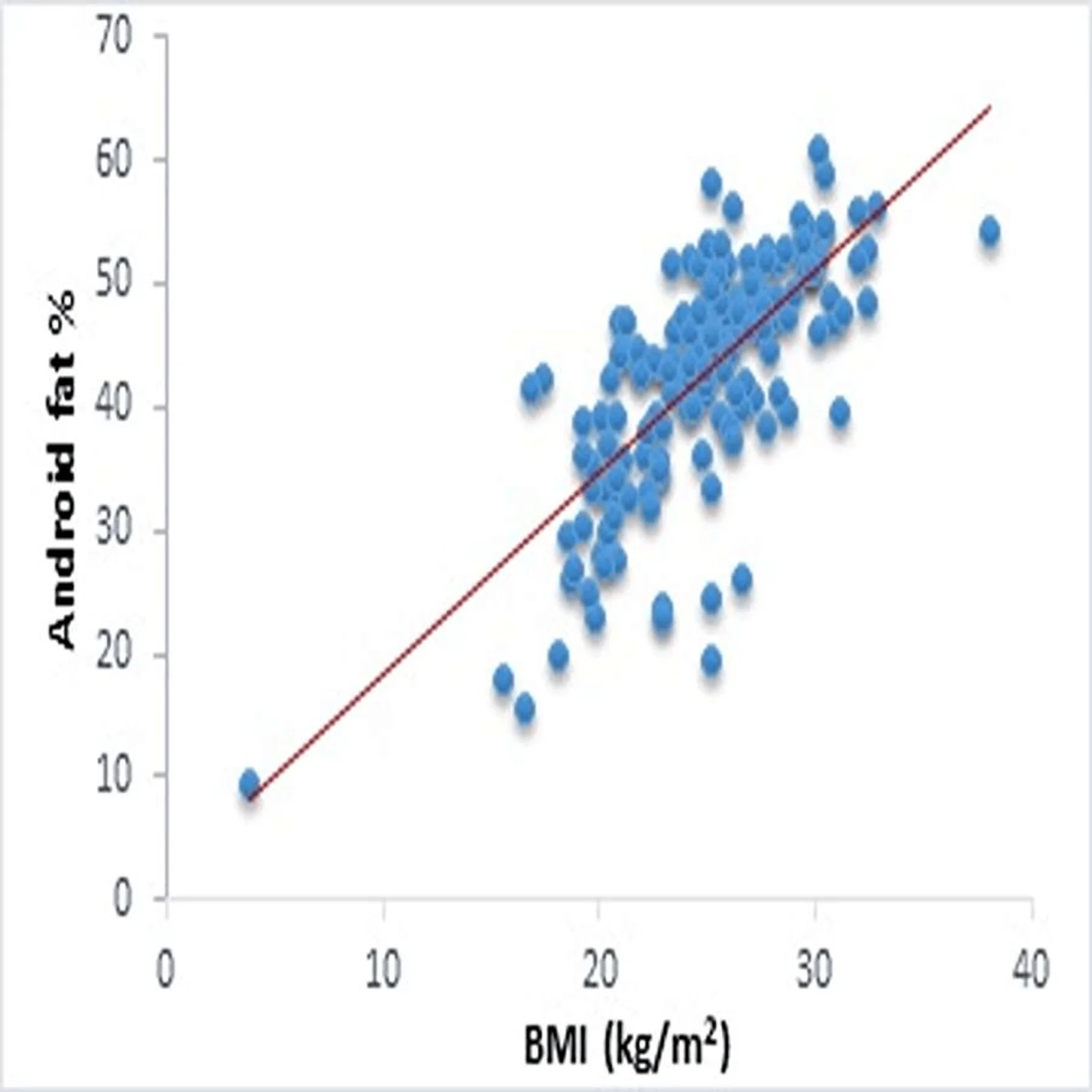
Graph showing the correlation between BMI (kg/m2) and android fat (%). BMI: body mass index

**Figure 10:**
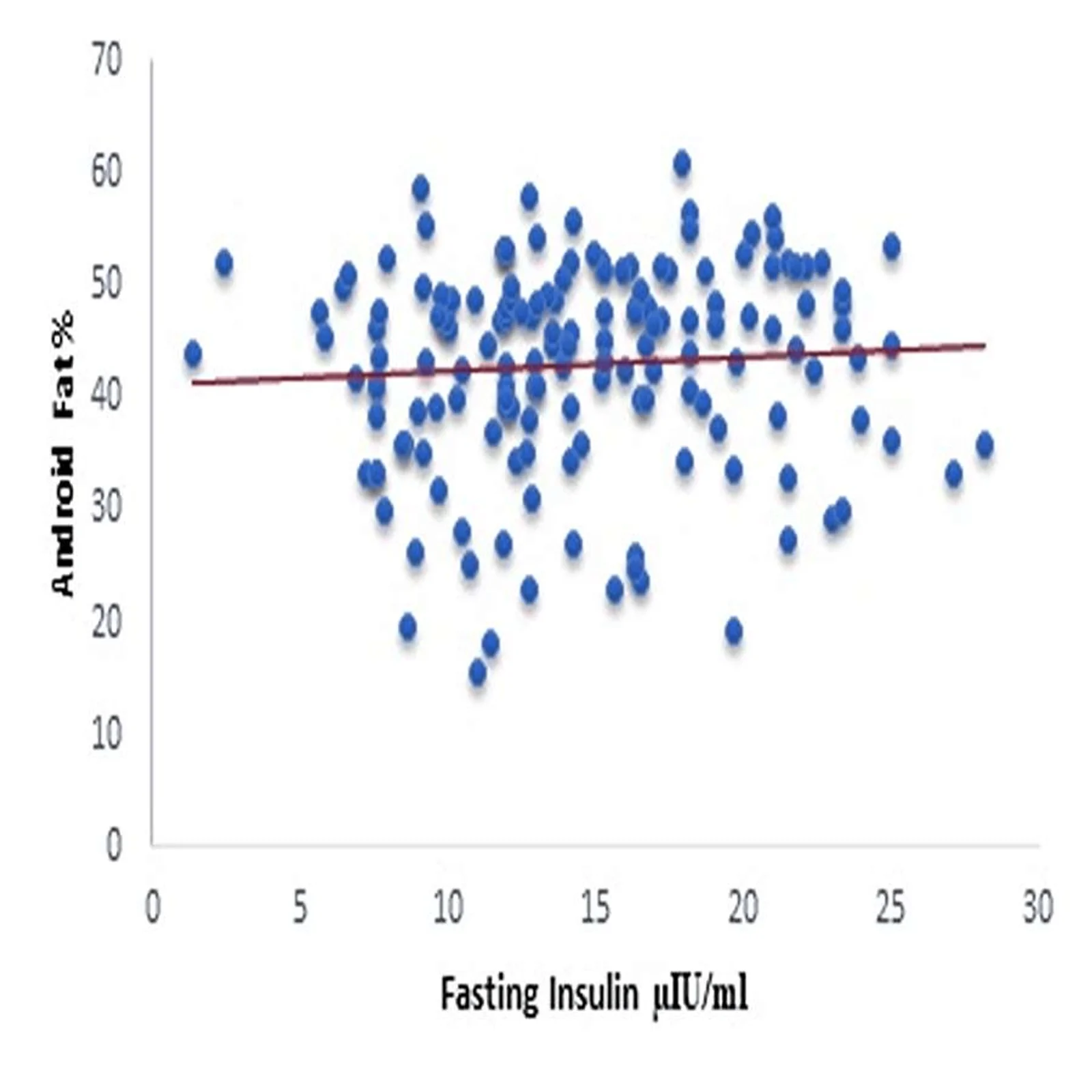
Graph showing the correlation between fasting insulin (µIU/mL) and android fat (%).

**Figure 11:**
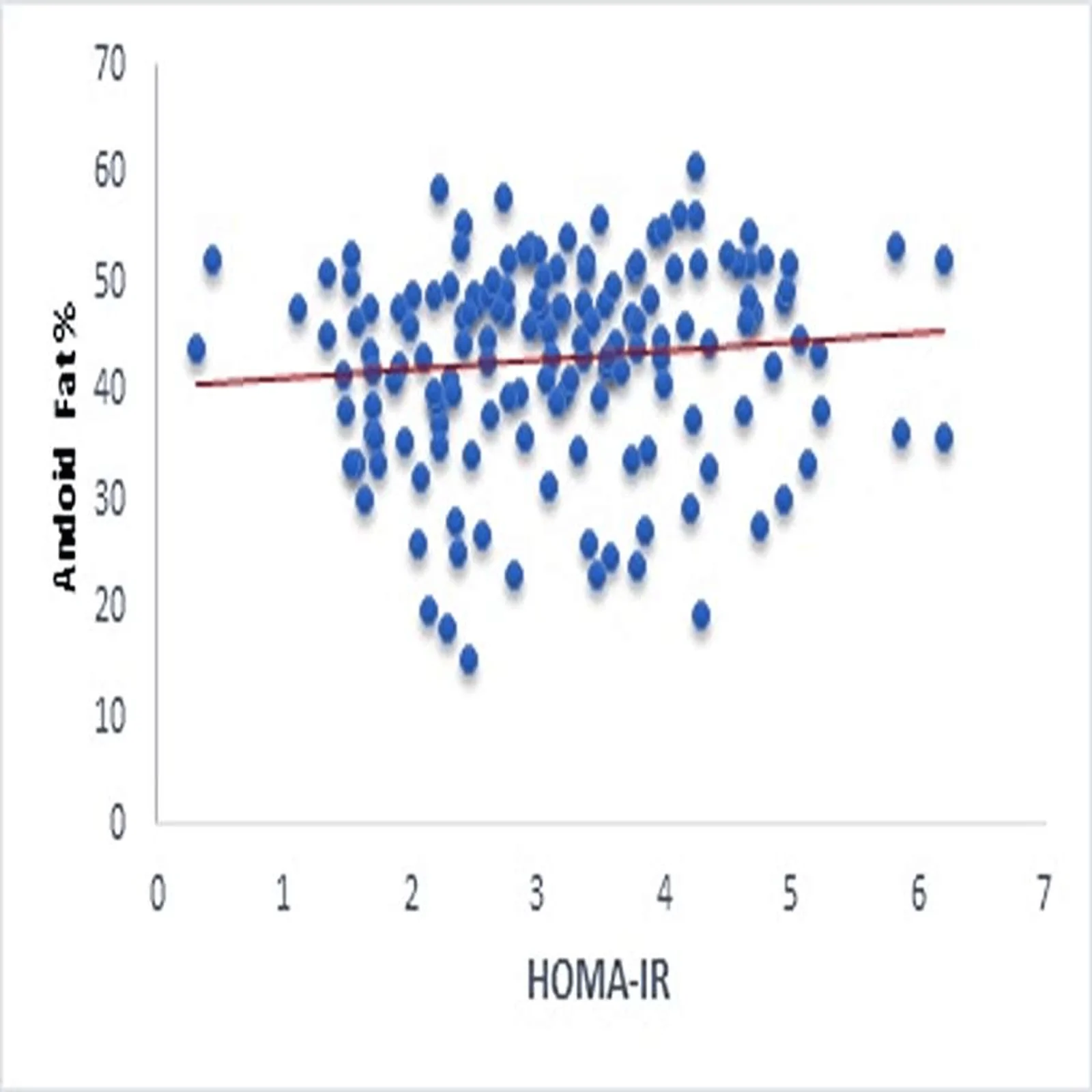
Graph showing the correlation between HOMA-IR and android fat (%). HOMA-IR: Homeostatic Model Assessment for Insulin Resistance

In correlation analysis, android fat presented significant positive correlations with BMI (r=0.697; p<0.001), fasting insulin (r=0.65; p=0.01), HOMA-IR (r=0.55; p=0.02), and WHR (r=0.497; p<0.001), while significant but weak association was observed with triglycerides (r=0.285; p=0.001), cholesterol (r=0.285; p=0.01), two-hour glucose (r=0.235; p=0.003), and FGS (r=0.264; p=0.001). Correlation analysis revealed no significant association between android fat and serum testosterone levels (r=0.061; p=0.45) (Table 4; Figure 8, Figure 9, Figure 10, and Figure 11).

Arm fat, leg fat, and gynoid fat demonstrated significant correlations with anthropometric variables rather than metabolic variables. Arm fat correlated significantly with BMI (r=0.536; p<0.001) and WHR (r=0.358; p<0.001), and leg fat (r=0.404; p<0.001) and gynoid fat (r=0.521; p<0.001), respectively, were significantly associated with BMI. Arm fat (r=0.058; p=0.47), leg fat (r=0.04; p=0.59), and gynoid fat (r=0.019; p=0.811), respectively, did not demonstrate a statistically significant association with serum testosterone levels. Further, no significant correlations were observed between arm fat and gynoid fat with the assessed insulin resistance markers (Table 4).

## Discussion

This study demonstrated the pattern of body fat distribution characterized by greater trunk and android fat accumulation among PMOS women; however, direct comparison with women without PMOS was not performed. Visceral fat accumulation has been consistently linked to insulin resistance and metabolic dysfunction, highlighting its pivotal role in the pathophysiology of PMOS [19]. Obesity, especially central obesity, is recognized as a major contributor to disease severity, and weight reduction remains a cornerstone of PMOS management, as emphasized by Della Corte et al. [20].

In the present study, android fat demonstrated significant positive correlations with BMI, fasting insulin, HOMA-IR, and WHR. Additionally, significant but weak associations were observed between android fat, two-hour glucose, triglycerides, total cholesterol, and FGS. These findings indicate that android fat deposition was significantly associated with metabolic disturbances and clinical hyperandrogenism (FGS) in women with PMOS. However, longitudinal studies are needed to clarify potential underlying mechanisms [8]. In support of our study, similar associations between android fat deposition, metabolic risk markers, and FGS have been reported in previous studies [21,22].

In this study, trunk fat significantly correlated with fasting insulin, BMI, HOMA-IR, and WHR. However, a significant but weak association was observed with triglyceride levels, cholesterol, two-hour glucose, and FGS. Earlier studies have also reported that trunk fat is closely related to triglyceride levels and other metabolic variables, reinforcing its clinical relevance as an indicator of metabolic risk in PMOS [23]. Although DEXA-derived adiposity measures demonstrated a statistically significant association with triglyceride levels, the magnitude of the association was weak.

Total body fat showed significant correlations with BMI and fasting insulin, while a significant but weak association was observed with WHR and FGS. These observations are consistent with findings from previous studies in women with PMOS. Several investigations using DEXA and other body composition assessment methods have demonstrated that higher total body fat is closely associated with insulin resistance, reflected by elevated fasting insulin levels. Studies have also reported modest relationships between total body fat and clinical manifestations such as central obesity and hyperandrogenism, measured by WHR and FGS. Collectively, these findings support the concept that excess total adiposity contributes to the metabolic and clinical features of PMOS, with stronger associations observed for metabolic parameters than for clinical markers of hyperandrogenism [24,25].

In the present study, both android and trunk fat demonstrated significant positive correlations with fasting insulin and HOMA-IR, with trunk fat showing the strongest associations. These findings suggest that greater android and trunk fat accumulation is associated with impaired insulin sensitivity in women with PMOS and are consistent with the established role of abdominal adiposity in the pathophysiology of insulin resistance. Visceral and upper-body fat are metabolically active depots that promote increased free fatty acid release, chronic low-grade inflammation, and altered adipokine secretion, thereby contributing to reduced insulin sensitivity. Similar associations between central adiposity, fasting hyperinsulinemia, and insulin resistance have been reported by previous studies [26]. However, because android and trunk fat were also strongly correlated with BMI in our cohort, these associations may partly reflect the influence of overall adiposity rather than an independent effect of regional fat distribution.

Gynoid, leg, and arm fat demonstrated significant associations, mainly with anthropometric indices such as BMI and WHR. Although gynoid fat contributes substantially to overall adiposity, its metabolic impact appears limited when compared with android fat, as also suggested by Aziz et al., who highlighted the android-to-gynoid ratio as a predictor of cardiovascular risk in PMOS [27]. Associations observed with arm and leg fat likely reflect overall adiposity rather than independent metabolic effects, consistent with prior reports [28,29]. Overall, our findings suggest the importance of the DEXA-derived assessment of regional fat distribution, particularly android, trunk, and total body fat, in identifying women with PMOS at increased metabolic risk [30].

An important consideration in interpreting our findings is the strong correlation between android fat mass and BMI. Therefore, the observed associations between android adiposity and metabolic parameters may partly reflect the influence of overall adiposity rather than an independent effect of regional fat distribution. As the present study did not include analysis adjusting for BMI, it was not possible to determine whether android fat provides additional predictive value beyond conventional measures of obesity. Future longitudinal studies employing multivariable models are needed to clarify the independent contribution of regional adiposity to metabolic risk in women with PMOS.

DEXA-derived assessment offers a more detailed characterization of body composition and regional adiposity compared with conventional anthropometric measures; however, the present analysis did not evaluate whether these measures provide incremental predictive value beyond BMI or WHR. Future prospective studies incorporating comparative predictive analysis and clinical outcome measures are warranted to establish whether DEXA-derived regional adiposity parameters provide additional clinical utility for risk stratification and management in women with PMOS.

Limitations

This study has several limitations. First, its cross-sectional design and the absence of a healthy control group limit causal inference and comparisons with normal body fat distribution; healthy controls were not included due to ethical concerns regarding unnecessary DEXA radiation exposure. Second, formal statistical comparisons between regional fat depots were not performed, as the study focused on evaluating the associations of individual DEXA-derived fat measures with metabolic and hormonal parameters. Third, participants were not stratified according to Rotterdam PMOS phenotypes, which may differ in metabolic and adiposity characteristics. Fourth, the DEXA scan was not standardized according to menstrual cycle phase, and the potential effect of cycle-related fluid variations on body composition measurements cannot be excluded. Finally, the analysis was primarily descriptive and based on unadjusted correlations; therefore, the findings should be considered exploratory rather than evidence of independent effects. Future studies incorporating matched controls, phenotype-specific analysis, and multivariable regression are needed to validate these findings.

Future perspective

This study underscores the importance of addressing body fat distribution in understanding the metabolic complexities of PMOS in Kashmiri women. However, several avenues for future research remain unexplored. Longitudinal studies are needed to establish causal relationships between changes in body fat distribution, insulin sensitivity, and metabolic parameters over time in this population. Such studies could provide deeper insights into the progression of metabolic complications in PMOS and the impact of interventions. Moreover, interventional studies focusing on lifestyle modifications, including dietary changes, physical activity, and emerging therapies such as pharmacological agents, should be conducted to assess their effectiveness in improving insulin sensitivity and reducing metabolic risks. Incorporating advanced imaging techniques to assess body fat distribution more precisely and using biomarkers to predict metabolic risks could enhance the accuracy of diagnosis and treatment in the future.

## Conclusions

DEXA-derived regional adiposity measures, particularly central fat depots, may be significantly associated with adverse anthropometric, insulin sensitivity, and metabolic parameters in women with PMOS. These findings suggest that central fat distribution may serve as a useful marker of metabolic risk and may help inform future risk assessment strategies. However, the present analysis does not establish that central adiposity contributes independently of overall adiposity. However, due to the cross-sectional design and absence of a control group, no conclusions regarding causality can be drawn. Given the unique genetic and environmental factors in the Kashmiri population, tailored interventions focusing on weight management, lifestyle modifications, and early detection of insulin resistance are crucial.
